# pH Gradients in Spatially Non-Uniform AC Electric Fields around the Charging Frequency; A Study of Two Different Geometries and Electrode Passivation

**DOI:** 10.3390/mi14091655

**Published:** 2023-08-23

**Authors:** Azade Tahmasebi, Sanaz Habibi, Jeana L. Collins, Ran An, Esmaeil Dehdashti, Adrienne Robyn Minerick

**Affiliations:** 1 Department of Chemical Engineering, Michigan Technological University, Houghton, MI 49931, USA; tahmaseb@mtu.edu (A.T.); jeanac@mtu.edu (J.L.C.); dehdasht@mtu.edu (E.D.); 2 Department of Chemical Engineering, University of Michigan, Ann Arbor, MI 48109, USA; sahabibi@umich.edu; 3 Department of Chemical Engineering, University of Houston, Houston, TX 77204, USA; ran@uh.edu

**Keywords:** pH gradient, dielectrophoresis, electrokinetic microfluidics, electrode passivation

## Abstract

Dielectrophoresis (DEP), a precision nonlinear electrokinetic tool utilized within microfluidic devices, can induce bioparticle polarization that manifests as motion in the electric field; this phenomenon has been leveraged for phenotypic cellular and biomolecular detection, making DEP invaluable for diagnostic applications. As device operation times lengthen, reproducibility and precision decrease, which has been postulated to be caused by ion gradients within the supporting electrolyte medium. This research focuses on characterizing pH gradients above, at, and below the electrode charging frequency (0.2–1.4 times charging frequency) in an aqueous electrolyte solution in order to extend the parameter space for which microdevice-imposed artifacts on cells in clinical diagnostic devices have been characterized. The nonlinear alternating current (AC) electric fields (0.07 V_pp_/μm) required for DEP were generated via planar T-shaped and star-shaped microelectrodes overlaid by a 70 μm high microfluidic chamber. The experiments were designed to quantify pH changes temporally and spatially in the two microelectrode geometries. In parallel, a 50 nm hafnium oxide (HfO_2_) thin film on the microelectrodes was tested to provide insights into the role of Faradaic surface reactions on the pH. Electric field simulations were conducted to provide insights into the gradient shape within the microelectrode geometries. Frequency dependence was also examined to ascertain ion electromigration effects above, at, and below the electrode charging frequency. The results revealed Faradaic reactions above, at, and below the electrode charging frequency. Comparison experiments further demonstrated that pH changes caused by Faradaic reactions increased inversely with frequency and were more pronounced in the star-shaped geometry. Finally, HfO_2_ films demonstrated frequency-dependent properties, impeding Faradaic reactions.

## 1. Introduction

Electrokinetic microfluidic devices are a versatile subcategory of lab-on-a-chip (LOC) platforms with promising separation, focusing, and detection attributes favorable for in situ applications in cellular and biomolecular diagnostics. LOC utilization in early clinical diagnostics leverages small sample volumes and reduced equipment infrastructure, typically providing more rapid results, with smaller material and energy requirements, leading to lower cost and increased flexibility, simplicity, and portability [1,2,3,4]. Despite operational and design advances, LOC devices display lower reproducibility and reliability than comparable bench-scale laboratory diagnostic tests [5,6]. Relatively recent research into ion gradient formation in small volume lower frequency nonlinear AC fields may help explain the losses in reproducibility and reliability [7,8,9,10]. Within the ~10^−2^ μL volumes present within microfluidic chambers, evolving media conditions can interfere with the bioparticles’ polarization [11,12,13]. To improve the reliability of electrokinetic microfluidic devices, we need a full understanding of the phenomena they exhibit and how to control them [14,15]. Thus, this work aims to provide a more complete understanding of the dynamically evolving pH changes that occur within electrokinetic microfluidic devices. Further, this work tests a possible operational control to enhance the dependability of these systems.

Within microfluidic devices, hydrodynamic [16] and electrical [17] forces have been extensively studied to manipulate biological particles or cells. Applying an electric field to aqueous cell suspensions within microfluidic devices can generate a variety of electrokinetic phenomena, including electrophoresis, electro-osmosis [18], diffusiophoresis [19,20], and dielectrophoresis [21]. The prevalence or absence of each phenomenon is dependent upon electric field characteristics (applied potential including direct current, alternating current or waveform, frequency, and amplitude), medium characteristics (conductivity, viscosity, and permittivity), and characteristics of the cell or biomolecule (charge expression, size, and polarizability).

Polarizable, dielectric particles or cells in a suspension exposed to nonuniform AC electric fields yield motion up or down electric field gradients, known as dielectrophoresis (DEP). Particle movement depends upon frequency-dependent relative polarizability properties, namely differences between the bioparticle’s complex permittivity and the complex permittivity of the medium solution [22,23,24].

Simultaneous to ion-influenced bioparticle polarizability and motion, electrochemical processes known as Faradaic reactions occur at the electrode surfaces embedded within DC and AC electrokinetic microfluidic microdevices [25,26]. Moreover, as the ratio of surface area to volume is high in microfluidic microdevices, electrical double layer (EDL) effects can have a greater influence on particle and analyte behaviors. Therefore, fully characterizing the EDL and Faradaic reaction behaviors within DEP device operation is necessary across a range of frequencies; prior studies explored fixed frequencies or limited ranges [27,28,29].

Electric double layers (EDLs) form at all aqueous interfaces within electrokinetic systems, including charged microdevice walls (PDMS, glass) as well as electrode metal interfaces; an EDL also exists around the polarizable bioparticle or cells [28,30]. Specific to electrode surface EDLs, Grahame demonstrated that ion reduction happens within the EDL rather than in the bulk solution. Additionally, the EDL attains equilibrium over the course of the electrode charging period, $t_{c}$ [31]:

(1) $$t_{c}=\frac{\varepsilon \;L}{\sigma \;\lambda_{D}}=\frac{1}{f_{c}}$$

where ε, σ, L, and${\;\lambda }_{D}$ denote the solution permittivity, solution conductivity, characteristic length, and Debye length, respectively. Within AC electric fields, the development and attainment of an equilibrium state depend upon the applied frequency. The electrode charging frequency predicts the timescale to establish the EDL; it is the inverse of $t_{c}$ [31,32]. The Debye length of monovalent ions within the medium is as follows [31]:

(2) $$\lambda_{D}=\sqrt{\frac{\varepsilon RT}{2F^{2}C_{0}}}$$

where R, T, F, and $C_{0}$ denote gas constant, temperature, Faraday constant, and bulk molar concentration, respectively [29,31,33].

The charging frequency, $f_{c}$, influences the establishment of the EDL. When the applied frequency is lower than the EDL’s $f_{c}$, ions have sufficient time to migrate to the surface such that the EDL is fully charged and established (the lower AC frequency limit yields the DC condition). In contrast, by applying frequencies larger than $f_{c}$, ions have insufficient time to migrate and align in the Stern and diffuse layers, thus the EDL can only partially charge and is not considered fully formed. This work explored frequencies at and very near $f_{c}$ in which the EDL is only partially developed [34,35], but was hypothesized to still influence ion behaviors within the microdevice.

In Faradaic current flow, electrons travel through the electrode–electrolyte interface as a result of electrochemical ion reduction or oxidation [36]. Prior foundational research has established that water electrolysis is the most prevalent Faradaic reaction in electrokinetic microfluidics with an aqueous medium [11,37,38]. Water electrolysis generates hydrogen and hydroxide ions per the redox reactions at electrodes as follows:

At cathode: 2H_2_O + 2e^−^ ↔ H_2_(g) + 2OH^−^ (3)

At anode: 2H_2_O ↔ O_2_(g) + 4H^+^ + 4e^−^ (4)

Faradaic byproducts, hydrogen and hydroxide ions, release into the limited volume within the microfluidic chamber; such ions impact pH in the vicinity of the electrode surface [38,39,40].

In previous work, micro-scale spatiotemporally controllable ion and pH gradients were generated using electrokinetic techniques in aqueous and methanol microfluidic environments; the gradients were quantified and attributed to Faradaic reactions and electromigration mechanisms [7,8]. Gradient artifacts were documented via human red blood cell (RBC) lysis [10] and crenation [9] under limited frequencies and amplitude conditions above $f_{c}$. Table 1 summarizes previously observed ion phenomena resulting from Faradaic reactions.

Table 1 illustrates that bioparticle motion and cell behaviors were studied in electric fields ranging from 0.02 to 0.2 V_pp_/μm and charging frequency ratios of 0.16${\;f}_{c}$ and 0.36 to 72 $f_{c}$. A knowledge gap exists in the parameter space from 0.16${\;f}_{c}$ to $0.9{\;f}_{c}$, with incongruent phenomena observed in the different frequency regimes.

Because Faradaic reactions at the electrode surface produce and release hydronium ions into the solution, it was hypothesized that physically isolating the electrodes could prevent or decrease the release of Faradaic reaction byproducts into the electrokinetic microfluidic chamber. Previous work demonstrated that hafnium oxide (HfO_2_) thin films provided thermal and chemical stability, optical transparency, and high dielectric constant [42,43,44]. Preliminary evidence over a limited frequency range (5 $f_{c}$–25 $f_{c}$) demonstrated HfO_2_ use as a passivation layer to impede unfavorable electrochemical reactions [45]. Thus, the impact of a passivation layer on Faradaic reaction activity was tracked by measuring pH gradient behaviors as a function of frequency below, at, and above $f_{c}$ with and without an HfO_2_ thin film.

In this study, 4 mM NaCl with a conductivity of 478 µS/cm and relative permittivity of 77.98 [46] was utilized in microdevices of two different geometries with a characteristic length of 100 µm. The Debye length and charging frequency were calculated to be 4.79 nm and 3.3 kHz, respectively. Thus, to fill the frequency range gap, frequencies of 0.2 $f_{c}$, 0.4 $f_{c}$, 1 $f_{c}$, and 1.4${\;f}_{c}$ at an electric field of 0.07 V_pp_/µm were explored in devices with planar T-shaped and star-shaped geometries, yielding spatially nonuniform electric fields with symmetry around single or multiple lines of reflection, respectively. The pH was quantified with a pH-sensitive fluorophore with respect to position, time, applied frequency, electrode geometry (T-shaped and star-shaped), as well as uncoated and 50 nm HfO_2_-coated electrodes. Calibrations and control experiments were conducted with the pH-sensitive fluorophore to account for photobleaching and fluorophore electromigration.

## 2. Material and Methods

### 2.1. Chemical and Sample Preparation

To optically measure pH within the microdevices, fluorescein-5-isothiocyanate (FITC ‘Isomer ‘I’) was prepared (Life Technologies, Carlsbad, CA, USA) in e-pure water (18.2 MΩ·cm, ultrapure water system, Thermo Fisher Scientific, Waltham, MA, USA) to a 0.2 M stock solution made using 155.57 mg FITC powder in 20 mL e-pure water. Stock aliquots were stored at −5 °C, thawed at room temperature for 30 min, and then serially diluted to 0.2 mM FITC for experiments. Diluted samples were stored at 5 °C and used within 12 h.

A 0.2 M NaCl (Macron Fine Chemicals, >99% pure, Radnor, PA, USA) solution was prepared to a conductivity of 478 μS/cm. NaOH and HCl were purchased from Sigma Aldrich, St. Louis, MO, USA. NaOH at 1 M was serially diluted to 0.01 M and 1 M HCl was serially diluted to 0.01 M, which were used to manually adjust pH for calibration experiments. All NaCl, NaOH, and HCl solutions were stored at 5 °C and used within two weeks. For calibration, 8 mL of 0.2 mM FITC and 8 mL f 0.2 M NaCl were mixed in 384 mL e-pure water to form 400 mL solutions with molar concentrations of 4 μM and 4 mM for FITC and NaCl, respectively. This solution was divided equally into five different dark glass containers and stored at 5 °C for less than a week. The solutions were equilibrated to room temperature prior to adjusting the pH to fixed pH values of 4, 5, 6, 7, 8, 9, and 10 by titrating in 0.01 M HCl or 0.01 M NaOH. A negligible additional volume was added, so 4 μM for FITC and 4 mM for NaCl were assumed to be the final concentration.

Solutions for microdevice experiments were prepared by mixing 1 mL of 0.2 mM FITC and 1 mL of 0.2 M NaCl in 48 mL e-pure water to form a 50 mL solution with final molar concentrations of 4 μM for FITC and 4 mM for NaCl. The pH of this solution was 7.7±0.2 and conductivity was 478 μS/cm.

### 2.2. Microdevice Design and Fabrication

**Figure 1:**
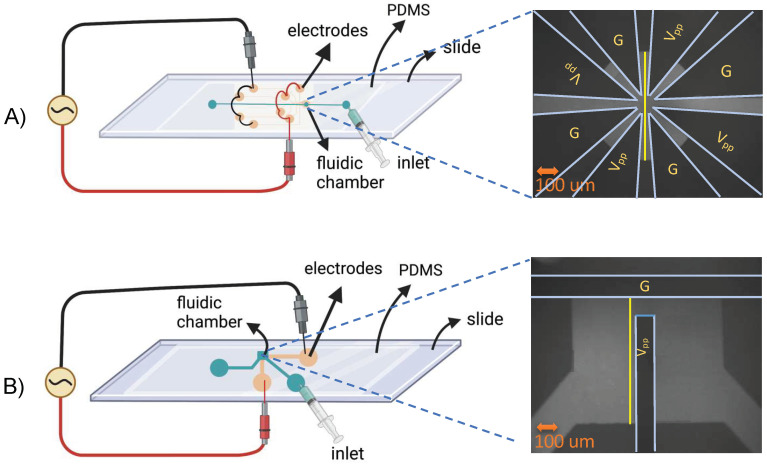
Star-shaped (**A**) and T-shaped (**B**) device diagrams with microscope images of the microfluidic chambers overlaying coplanar electrodes. FITC solutions were injected into the fluidic chamber and the device was mounted on the microscope stage with AC potentials applied to the electrodes. The yellow vertical lines added to the images indicate intensity analysis locations (figure components were created with BioRender.com, accessed on 15 June 2023).

Two Cr/Au microelectrode patterns were photolithographically patterned onto glass microscope slides (AmScope, Irvine, CA, USA). As shown in Figure 1, the T-shaped microelectrodes were perpendicularly positioned with a 100 μm gap. The star-shaped design had four triangular pairs of microelectrodes positioned symmetrically in a circle with a central gap of 100 um. Both planar electrode patterns were fabricated with 15 nm Cr/95 nm Au following standard photolithography, metal deposition, and lift-off techniques [47]. Half of the fabricated slides were coated with 50 nm HfO_2_ as a dielectric film following a previously reported electron sputtering technique [42].

For the microfluidic layer, degassed polydimethylsiloxane (PDMS, Dow, Midland, MI, USA) at a 10:1 monomer/curing agent ratio was cast over a Si/SU-8 wafer patterned with 150 μm fluidic channels and a 900 μm × 700 μm or 600 μm diameter chamber for T-shaped and star-shaped devices, respectively. A 3 mm biopsy punch formed fluidic ports. The PDMS castings and glass slides with microelectrodes were surface treated in a plasma chamber (Harrick Plasma Inc., Model # PDC-001, Ithaca, NY, USA), followed immediately by stereoscope alignment of the fluidic layer over the electrodes to form a PDMS/glass water-tight bond. Connectors for the syringe were secured at the inlet and outlet using clear epoxy. Wire leads were secured to the exposed electrode pads on the glass microscope slide using conductive silver epoxy (MG Chemicals, Burlington, ON, Canada); both were allowed to cure for 1 h.

### 2.3. Experiments and Data Acquisition

*pH Intensity Calibration:* The pH calibration solutions (ranging from 4 to 10) were injected into a 600 μm diameter microfluidic chamber bonded to a clear glass slide without electrodes; the fluorescent intensity of each solution was observed for 120 s via a fluorescent Zeiss Axiovert Microscope at 10X magnification and a fixed exposure time of 308.4 ms. All experiments were repeated five times using the same stock pH solution; pH values were verified and readjusted as necessary just prior to experiments (further details in [47]).

*Controls:* Control parameters utilized universally across all electric field experiments included a fixed initial pH of 7.7. For calibration controls, pH solutions were intentionally adjusted. Solution concentrations were fixed to 4 μM FITC and 4 mM NaCl with a conductivity of 478 μS/cm. Microscope magnification and exposure time were fixed at 10X and 308.4 ms, respectively. Applied electric fields in both devices were consistently 0.07 V_pp_/μm. In addition, strategic numerical normalizations were utilized to effectively compare experiments. These solution and AC electric field conditions are the same order of magnitude as prior FITC electromigration (0.055 V_pp_/µm, conductivity of 480 μS/cm) [45]. Prior control experiments yielded undetectable charged molecule electromigration in both uniform and non-uniform electric fields over time [7,45]. Electromigration was not observed initially, nor over time, indicating that non-Faradaic current-driven capacitive charging was negligible. Thus, the present work focused upon Faradaic reaction effects [7,8,45].

*Observation of Faradaic Reaction Byproducts and pH Gradients:* The 4 μM FITC and 4 mM NaCl solution to measure pH was brought to room temperature and injected into the T-shaped and star-shaped microfluidic chambers utilized to study temporal and spatial pH gradients. Devices were placed on the microscope stage, and an alternating current (AC) potential from a function generator was applied via the electrode leads. The FITC intensity within the fluidic chamber was observed at 10X and 308.4 ms exposure time using a 485 ± 25 nm filter. Fluorescent images were recorded for 120 s at a rate of one frame every 3 s, which resulted in 40 frames per experiment. The peak-to-peak voltage was kept constant at 0.07 V_pp_/μm, and frequencies of 660 Hz (0.2 $f_{c}$), 1.98 kHz (0.6 $f_{c}$), 3.3 kHz (1 $f_{c}$), and 4.62 kHz (1.4 $f_{c}$) were applied. The AC signal was applied after the first two frames. Experiments were repeated five times with flushing and reloading of the microdevice with the same FITC/NaCl stock solution.

Microdevices were flushed with e-pure water after experiments and for overnight storage. Prior to each new set of experiments, microdevices were examined visually at 10X for any electrode degradation or wear.

#### pH-Gradient Analysis

Microscope images contained spatial intensity data as a function of time. A Python script was developed to import the image files (Tifffile library) and compute the intensity as a function of time and location. A linear analysis region (see yellow lines in Figure 1) was defined within the grayscale image to analyze FITC intensity over time and position. For the T-shaped geometry, a vertical line adjacent to the vertical electrode and for the star-shaped geometry, a vertical line in the center of the fluidic chamber were utilized, and the intensity over time (120 s) and position for different frequencies were acquired. An identical analysis was followed for the HfO_2_-coated devices.

Similar to the pH calibration, intensity magnitudes were normalized by dividing the intensity at each time point by the initial intensity (*t* = 0) before the electric field was applied to the system $\left(i_{N}=\frac{i_{t}}{i_{t=0}}\right)$. In addition to controlling the exposure time, solution concentration, initial pH, and initial solution conductivity, this normalization was used to increase the reliability of the comparison between experiments. The normalized intensity was converted to pH using the pH–intensity Equation (5).

To determine the statistical significance of the pH differences at different experimental conditions, two-factor replication and pair-wise analysis of variance (ANOVA) calculations were computed using Excel’s built-in functions.

### 2.4. Simulations

Comparisons between the observed results and the electric field gradient simulations were carried out using COMSOL Multiphysics 5.4: Electric Currents physics with a frequency domain study. The simulations were run on Michigan Technological University’s High Performance Computing Shared Facility (Superior). The physical device geometries were identical to Figure 1. Hafnium oxide film thicknesses of 0.01 μm were utilized, consistent with microfabricated films; further, the HfO_2_ thickness along the vertical sides of the electrodes was treated as 20% of the horizontally deposited hafnium oxide layer. The electrode potential was set to be −7 V (ground) and 7 V (V_pp_), with a frequency of 4620 Hz (1.4 $f_{c}$). All other chamber and surface boundaries were set as electrical insulation. Material properties are shown in Table 2 and were obtained from COMSOL’s material library, experimental values, and previously characterized films deposited in the same facility [42]. Qualitative comparisons were performed between the experimental and simulation results.

## 3. Results and Discussion

The impacts of four independent variables on spatial pH changes were explored: time from electric field application, frequency, electrode geometry, and the presence/absence of the HfO_2_ passivation layer. Four different frequencies were tested below, at, and above the electrode charging frequency, including 660 Hz (0.2 $f_{c}$), 1.98 kHz (0.6 $f_{c}$), 3.3 kHz (1 $f_{c}$), and 4.62 kHz (1.4 $f_{c}$), at the fixed parameters previously described.

### 3.1. pH–Intensity Calibration and Photobleaching

Calibration of individual pH solutions from 4 to 10 was conducted according to the procedure described previously. Five repeats for each pH were averaged by time stamp. Images were imported to Python, cropped to the 600 μm circle, and processed to collect intensity data over time for each pH solution. Five repeats for each pH were averaged by time stamp; in addition, a mean intensity and standard deviation for each pH was obtained. FITC time curves across pHs were combined by normalizing by the initial intensity $\left(i_{N}(t)=\frac{i_{pH}(t)}{i_{pH@t=0}}\right)$. The normalized pH–intensity calibration curve is available in the Supplemental Information. The normalized intensity ($i_{N}$) versus pH was approximated with a sigmoidal function [48] with four parameters obtained using curve-fitting. The following equation was subsequently used to convert normalized intensity to pH:

(5) $$pH=0.79-\frac{1}{-11.95}ln\;\left(\frac{0.61}{1.06-i_{N}}-1\right)$$

The average pH by time stamp was examined to assess photobleaching, a fluorophore molecules’ diminishing ability to fluoresce as excitation time increases [49]. FITC’s emission declined by only 1.4% of the initial intensity over 120 s (graph in SI) and was deemed negligible compared with the 15–17% changes in intensity observed during electric field pH experiments. Therefore, photobleaching was neglected in all further analyses.

### 3.2. Time Dependency

Upon applying the electric field to the microfluidic device loaded with the pH-sensitive FITC fluorophore (initial pH = 7.7 and conductivity = 478 μS/cm), the emission intensity was observed to change within the microfluidic chamber over time. Per the calibrations and controls, increases in light emission intensity correspond to higher pH values. The qualitative results are first discussed, then conversions into quantified pH are demonstrated and discussed.

**Figure 2:**
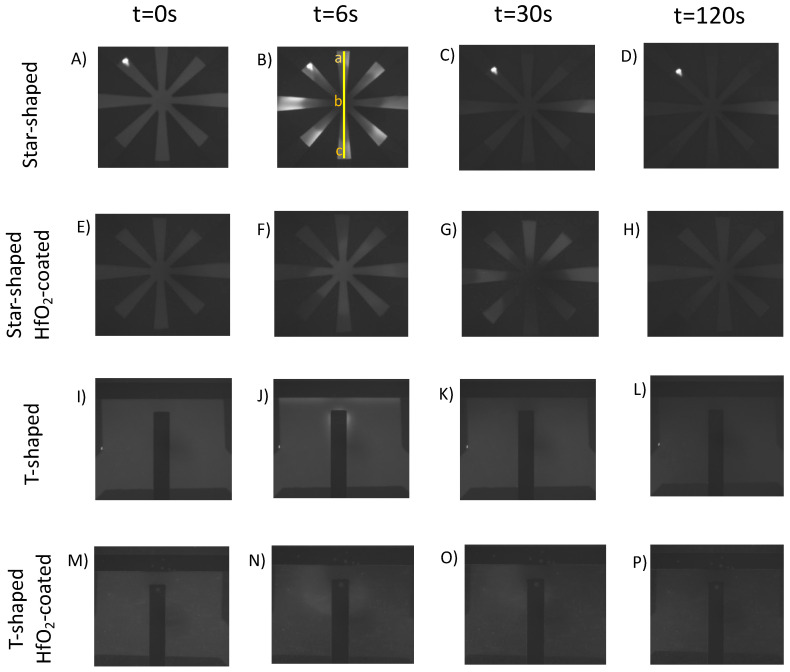
Change in FITC intensity, which is indicative of pH, is shown for star-shaped and T-shaped microfluidic devices with and without an HfO_2_ layer at 0.6 $f_{c}$ and 0.07 V_pp_/μm. Rows are organized as star-shaped (**A**–**D**), star-shaped HfO_2_-coated (**E**–**H**), T-shaped (**I**–**L**), and T-shaped HfO_2_-coated (**M**–**P**). Columns are organized by time: the beginning of the experiments (*t* = 0 s), 1 s after applying an electric field (*t* = 6 s), *t* = 30 s, and experiments end (*t* = 120 s).

Figure 2 shows comparative qualitative images of experiments for the T-shaped and star-shaped devices at 0.6 $f_{c}$ (1.98 kHz) and 0.07 V_pp_/μm. Rows from top to bottom illustrate star-shaped uncoated, star-shaped HfO_2_-coated, T-shaped uncoated, and T-shaped HfO_2_-coated; columns from left to right illustrate the FITC intensity at time 0 s (no electric field), 6 s (1 s after the electric field was applied), 30 s, and 120 s. At time 0, the FITC intensity was uniform throughout the microfluidic chambers (disregarding contamination at the top left of the uncoated star-shaped device). After applying an electric field, at *t* = 6 s, a rapid increase in FITC intensity radially propagating from the star center was observed; this dramatic burst in intensity lasted 2–3 s, then the intensity decreased uniformly and slowly across the microfluidic chamber, as illustrated at *t* = 30 s. By 120 s, the intensity was lower than the initial conditions. A comparison between the uncoated and HfO_2_-coated device in the second row shows a substantially attenuated FITC intensity response both with the initial intensity burst and over the entire 120 s. At *t* = 6 s, the HfO_2_-coated device did not display as intense or as rapid of spatial propagation of the intensity burst. Further, the decline in FITC intensity in the star-shaped HfO_2_ device had a smaller pH shift and declined at a slower rate than in the uncoated device.

The third and fourth rows in Figure 2 illustrate similar qualitative phenomena in the T-shaped microfluidic device; a rise in intensity was observed at high field areas adjacent to the electrodes. However, the magnitude of the short-term intensity increase was less than what was observed in the star-shaped device. Further, the HfO_2_-coated T-shaped device followed a similar trend whereby the FITC intensity change was attenuated compared with the uncoated device.

Collectively, these images qualitatively illustrate a short timescale burst in intensity corresponding to a rise in FITC intensity at the highest electric field regions followed by a longer timescale decline in FITC intensity below the starting value. Further, the impact of the HfO_2_ passivation layer was observed; the passivation layer attenuates the pH shifts (FITC intensity increases and decreases) within the solution.

**Figure 3:**
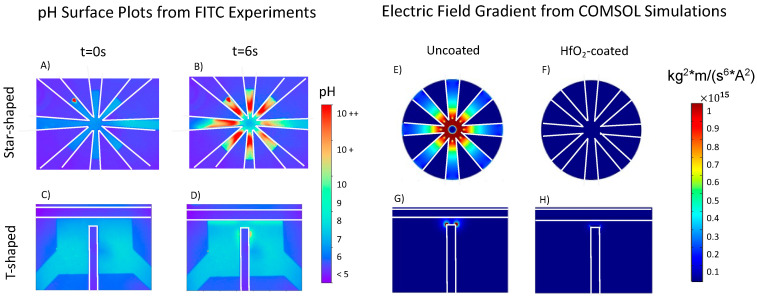
Quantified pH surface plots of star-shaped (**A**,**B**) and T-shaped (**C**,**D**) uncoated devices at 1.4 $f_{c}$ and 0.07 V_pp_/μm. The pH was fairly uniform in both geometry devices around 7.7; pH increased rapidly upon applying an electric field. This burst in pH was much more pronounced in the star-shaped devices. The electric field gradient from COMSOL simulations for uncoated (**E**,**G**) and coated (**F**,**H**) devices for both geometries. The simulated electric field gradients in both uncoated geometries show similarities in shape with regions of high pH in the respective experiments.

The FITC pH–intensity calibration sinusoidal fit Equation (5) was utilized to ascertain quantitative changes in pH. The short timescale bursts in intensity, as illustrated in Figure 2B,J for star-shaped and T-shaped, respectively, were further explored by converting FITC intensity values to pH to generate pH surface plots. Figure 3 illustrates this for 1.4 $f_{c}$ and 0.07 V_pp_/μm. The increasing pH burst in the star-shaped device is symmetric through the fluidic chamber, while in the T-shaped device, the pH burst occurred near the top of the vertical electrode and in areas adjacent to the horizontal electrodes. These rapid bursts were more pronounced in both uncoated device geometries above the charging frequency; the burst intensity increases with the increasing frequency.

The star-shaped device has eight electrodes each with an exposed surface area within the chamber yielding a total area of 0.155 mm^2^, while the T-shaped electrode device has an exposed electrode surface area of 0.105–0.114 mm^2^. Thus, the exposed T-shaped electrode surface area is 67–74% less than the star-shaped electrode surface area. Both device geometries have a minimum electrode gap of 100 μm, but the electrode perimeter proximity is maximized in the star-shaped device compared with the T-shaped design, as illustrated by the electric field gradient simulations in Figure 3E,G. Thus, it was postulated that the more pronounced burst in pH over short timescales in the star-shaped devices compared with the T-shaped device was because the electrode surface area, which catalyzes Faradaic reactions, is greater.

Because Faradaic reactions occur only at the electrode surface, apparent reaction rates are subject to passive mass transfer of water diffusion to the surface and ion diffusion away from the surface and into bulk of the ~10^−2^ μL chamber. Consistent with Equations (3) and (4), the hydronium and hydroxide ions are products of the reduction and oxidation reactions; both ions impact the pH adjacent to the electrode. For each electron transferred with the applied current, one H^+^ and one OH^−^ are produced [38,39]. However, the reaction rates are not identical; in water electrolysis, the oxidation rate surpasses the reduction [50,51,52]. This disparity arises from the fact that the oxidation reaction is thermodynamically more favorable compared with the reduction reaction. In the oxidation half-reaction (4), electrons are lost, while in the reduction half-reaction (3), electrons are gained. Consequently, the oxidation reaction is exothermic, releasing energy, whereas the reduction reaction is endothermic, requiring energy input. Thus, energy favors oxidation, assuming all other factors remain constant [52].

Further, oxidation reactions lose electrons, while reduction reactions gain electrons to form a new bond with another atom. Thus, the formation of a new bond is kinetically slower, such that oxidation reactions occur more quickly than reduction reactions [50,51]. For the Faradaic reactions in this system, this results in a higher production of H^+^ compared with OH^−^ over longer timescales, which causes a steady decline in the pH of the solution.

Further, the ion electromobility is determined by a charge-to-size ratio. H^+^ and OH^−^ have the same, but opposite charge, with different sizes. Hydronium molecules (once H^+^ is complexed with water) have a molar volume of −5.4 cm^3^/mol [53] and are larger than hydroxide ions, with a molar volume of 1.2 cm^3^/mol [53]. This results in slower hydronium and faster hydroxide electromigration. Hydroxide’s greater mobility within the alternating electric field causes a rapid increase in OH^−^ and thus pH value, manifesting as an intensity burst from the high field regions in the first 6 s of electric field application. At longer timescales, the pH decreases because the rate of hydrogen ion production is greater and gradually overpowers the finite ~10^−2^ μL volume in the microchamber.

At cathode (reduction, slower): 2H_2_O + 2e^−^ ↔ H_2_(g) + 2OH^−^

At anode (oxidation, faster): 2H_2_O ↔ O_2_(g) + 4H^+^ + 4e^−^

**Figure 4:**
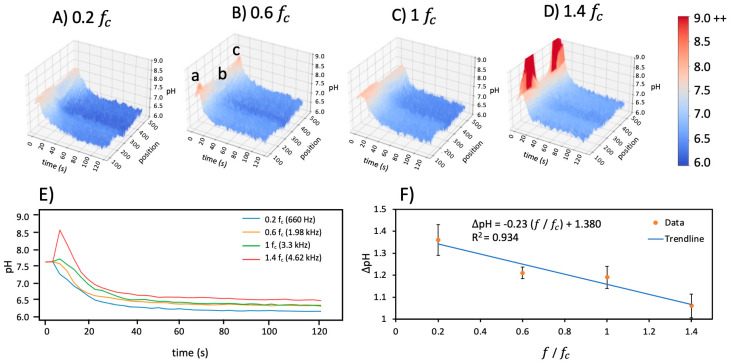
The pH as a function of time and position for the uncoated star-shaped microdevice at (**A**) 0.2 $f_{c}$, (**B**) 0.6 $f_{c}$, (**C**) 1 $f_{c}$, and (**D**) 1.4 $f_{c}$. Positions a, b, and c in (**B**) correspond to the same positions in Figure 2A. (**E**) pH over time for the four frequencies explored. A pH increase or burst in the short timescale is observed above the charging frequency, but the pH in the long timescale lowers as charging frequency decreases. (**F**) The change in pH (∆*pH*) between 0 s and 120 s is plotted versus the normalized frequency with a linear trendline; data points are statistically significant at *p* < 0.05.

To enable quantitative pH gradient comparisons between experimental parameters, 3D graphs of pH, position, and time were plotted for both geometries using the profile lines noted from a to c in Figure 1. Figure 4 shows the 3D graph from the line profile in uncoated star-shaped microdevices averaged over five experiments for four $f_{c}$ frequencies at 0.07 V_pp_/μm. This 3D representation quantitatively illustrates the short-term increase in pH followed by the gradual decrease in pH over the 120 s experiment. Spatial variations are also apparent, with the lowest pH occurring near the central singularity of the star shape and remaining over the entire 120 s.

### 3.3. Frequency Dependency

The pH gradient was also examined and quantified as a function of frequency for 0.2 $f_{c}$, 0.6 $f_{c}$, 1 $f_{c}$, and 1.4 $f_{c}$. The initial pH and conductivity were 7.7 and 478 μS/cm, respectively. The pH increased at all frequencies in the short term, then declined over 120 s to settle at values less than the initial pH conditions. These behaviors were most severe above the charging frequency, as illustrated in Figure 4D for the star-shaped microdevices.

Corresponding 2D plots of pH over time were acquired by taking an average of the position dimension. Figure 4E shows the averaged pH over time for each frequency and demonstrates the intensity/pH burst in the first few seconds followed by a decrease in pH over longer timescales. The short timescale rise in pH is most pronounced above the charging frequency at 1.4 $f_{c}$; this peak becomes increasingly less prominent at frequencies below the charging frequency.

Examining the longer timescale of pH demonstrates a different trend. Figure 4F presents the change in pH ($∆pH={pH}_{t=120}-{pH}_{t=0}\;$) from 0 s to 120 s. The ∆pH decreases with the increasing frequency. This suggests that the effects of the hydronium products from the Faradaic reactions are more pronounced at lower frequencies below the charging frequency, while hydroxide ions with greater electromobility play a larger role above the charging frequency. A linear trendline was fit to the change in pH versus normalized frequency, $\frac{f}{f_{c}}$, showing a negative slope of 0.23 with an R^2^ of 0.93.

A two-factor with replication ANOVA yielded an F value greater than the F_critical_ value for a *p* of 0.05, which revealed that frequency had a statistically significant impact on the pH change for both star-shaped and T-shaped geometries.

### 3.4. Geometry Dependency

**Figure 5:**
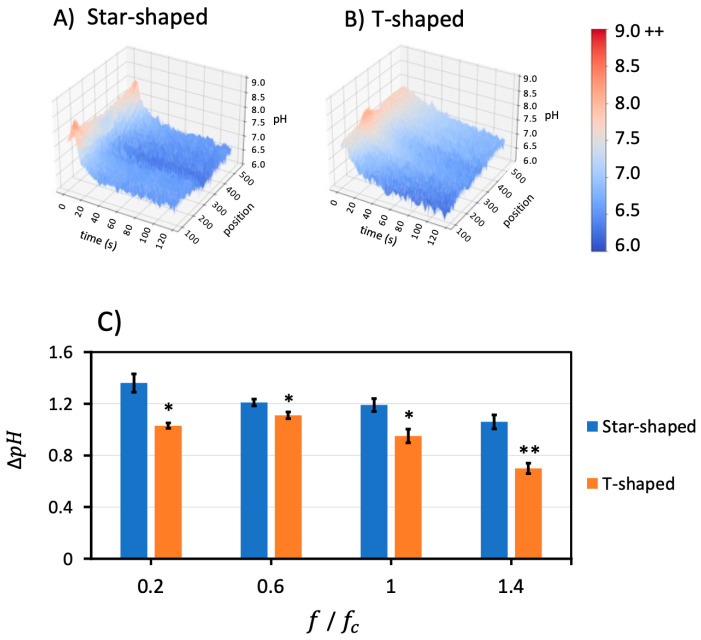
Geometry comparison of pH over time and position for (**A**) star-shaped and (**B**) T-shaped geometries when applying 0.6 $f_{c}$ (1.98 kHz) frequency and 0.07 V_pp_/μm. The highest pH was at larger radial positions in the star-shaped device. The maximum pH in the T-shaped device occurred closest to the tip of the vertical electrode. (**C**) Comparison of the $∆pH={pH}_{t=120}-\;{pH}_{t=0}\;$for star-shaped and T-shaped devices for different frequencies. Whisker bars indicate standard deviation. Pair-wise ANOVA results are indicated for * *p* < 0.05 and ** *p* < 0.001 between geometries. Further, ∆*pH* of the T-shaped device is always less than that of the star-shaped device.

Two geometries were utilized to produce nonuniform electric fields: star-shaped and T-shaped. As shown in Figure 1, the star-shaped device had eight triangular electrodes with an exposed area of 0.155 mm^2^, while the T-shaped device had two electrodes with an exposed electrode surface area of 0.105–0.114 mm^2^. Differences between the two geometries include single versus multiple axes of symmetry, different net spatial proximity between the 0.07 V_pp_/μm and ground electrodes, as well as total exposed electrode surface area within the microchamber. Figure 5 demonstrates the 3D representation of fluorescently measured pH at different positions over time for both geometries at a fixed frequency of 0.6 $f_{c}$ (1.98 kHz). The star-shaped geometry in Figure 5A reveals that the outer regions of the star-shaped geometry demonstrate the highest pH, corresponding to the burst in OH^−^ at *t* = 6 s, 1 s after the electric field was applied (same conditions as Figure 2B). The intensity burst in the T-shaped device was smaller and discernable in the vertical profile line (Figure 5B) near the tip of the vertical electrode (Figure 2J).

A quantitative pH comparison was conducted between geometries at different frequencies. The short-term burst behavior in the T-shaped devices tended to be 85% smaller than for the star-shaped at frequencies above the charging frequency. The initial to final pH change, ∆*pH*, revealed that the T-shaped device was 60–90% of the star-shaped device. This suggests that the shorter timescale influence of hydroxide ions and longer timescale influence of hydronium ions from the Faradaic reactions were likely consistent between electrode geometries, but the differing electrode surface area (0.105–0.114 versus 0.155 mm^2^) between the designs accounts for the attenuated responses in the T-shaped versus star-shaped electrode designs. It is interesting that the T-shaped electrode surface area is 67–73% of the star-shaped surface area, and this is roughly the order of magnitude reduction in the total pH change of the T-shaped device over the course of the experiment.

To visualize the impact of geometry on ∆*pH*, bar charts were compiled as shown in Figure 5C. To explore pair-wise statistical significance, ANOVA was performed to explore whether the geometry factor statistically affected ∆pH at all four different frequencies. The results showed that F > F_critical_ for the geometry factor, which confirms statistical significance at *p* < 0.05 and *p* < 0.001, as noted in the figure.

### 3.5. Passivation Layer Impact

**Figure 6:**
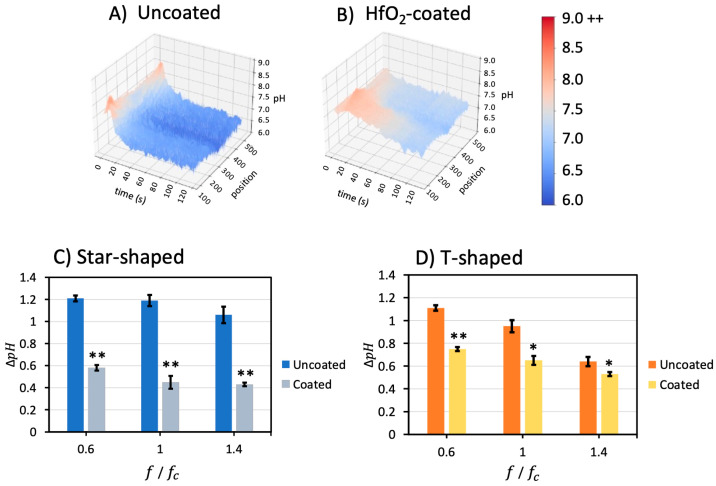
Comparison between uncoated (**A**) and coated (**B**) star-shaped devices at 0.6 $f_{c}$ (1.98 kHz) and 0.07 V_pp_/μm. The pH decreased from 7.7 to around 6.5 in (**A**); however, the pH in (**B**) decreased from 7.7 to around 7.0. Thus, the hafnium oxide layer reduced the Faradaic reaction rate. (**C**) The ∆*pH* for the star-shaped HfO_2_-coated device was reduced by an average of ~60% compared with the uncoated device. (**D**) The ∆*pH* for HfO_2_-coated T-shaped devices was reduced by ~30% on average. Coated/uncoated electrodes were treated as an independent factor in ANOVA; coating has a significant effect on ∆*pH* in both geometries at the designated *p*-values of * *p* < 0.05 and ** *p* < 0.001.

A final dependency, a hafnium dioxide (HfO_2_) passivation layer sputter deposited on top of the electrodes, was explored as a mechanism to eliminate or reduce Faradaic reactions. The pH gradient was measured for both geometry devices coated with 50 nm of HfO_2_. Figure 6A,B illustrate 3D pH, time, and position profiles for uncoated and coated star-shaped devices when applying 0.6 $f_{c}$ (1.98 kHz) and 0.07 V_pp_/μm. The short timescale burst and long timescale ∆pH was substantially lower in the HfO_2_-coated devices. In Figure 6A, the pH decreased from 7.7 to around 6.5; however, the pH in the HfO_2_-coated device in Figure 6B only decreased from 7.7 to around 7.1.

The ∆*pH* change from the beginning to the end of the experiments was calculated for uncoated and coated devices for different frequencies and compiled in Figure 6C,D. The pH change was reduced by ~60% for the star-shaped device coated with an HfO_2_ layer for 0.6 $f_{c}$, 1 $f_{c}$, and 1.4 $f_{c}$. For coated T-shaped devices, the pH change was reduced by ~30% for the same frequencies. The experiments for 0.2 $f_{c}$ were repeated only once because this low frequency caused irreparable damage to the electrodes. Unfortunately, this limited the ability to reproduce the results from Sanchez [32,33] at 0.167 $f_{c}$.

Coated/uncoated electrodes were treated as an independent factor in ANOVA, with the result that coating has a significant effect on ∆*pH* in both geometries. These results demonstrate that the HfO_2_ layer impedes electron transfer between the electrolyte and the metal surface, thus decreasing the extent of the Faradaic reactions at the electrode surface, limiting OH^−^ and H^+^ ions released into the solution and attenuating pH shifts in both the short timescale as well as the long timescale. As such, Faradaic reaction byproducts below, at, and above the electrode charging frequencies in two geometries of electrokinetic microfluidic devices can be significantly reduced with a hafnium dioxide passivation layer.

## 4. Conclusions

This work utilized a pH-sensitive fluorophore to qualitatively and quantitatively observe pH gradient spatial and temporal phenomena in two nonlinear electrokinetic microfluidic devices. Two spatially nonuniform microelectrodes with T-shaped and star-shaped geometries were adopted to create nonlinear electric fields with single and multi-axis symmetry. A 4 mM NaCl solution with 478 μS/cm conductivity was utilized across all experiments, whereby the calculated Debye length was 4.79 nm, yielding an electrode charging frequency, $f_{c}$, of 3.3 kHz. Experiments were conducted at 0.2, 0.4, 1, and 1.4 times $f_{c}$ and at a constant 0.07 V_pp_/μm electric field with and without a hafnium dioxide (HfO_2_) passivation layer coating of microelectrodes.

Experiments were conducted for 120 s, and the pH gradient was observed over time. After careful photobleaching and electromigration controls, the intensity of FITC was utilized as an indicator of pH value or H^+^ concentration spatially and temporally. The intensity increased suddenly immediately after applying an electric field, most prominently adjacent to areas with the highest electric field density, and then decreased over time in both T-shaped and star-shaped devices. The sudden rise in pH upon applying an electric field was most apparent for frequencies above and at the electrode charging frequencies for both device geometries. However, this peak was most prominent in the star-shaped devices at 1.4 $f_{c}$. Comparisons between different frequencies revealed that the overall experiment ∆*pH* change decreased with the increasing frequency. This trend was observed for both geometries. These changes in FITC intensity, and thus pH, revealed the presence of undesired Faradaic reactions (water electrolysis) below, at, and above the electrode charging frequency. An unexpected but insightful result revealed that smaller hydroxide ions from Faradaic reactions have greater electromobility and appear to dominate the pH response in the shorter timescale, with an intensity burst corresponding to an increase in pH. At longer timescales, hydronium ions from Faradaic reactions are produced at a higher rate compared with hydroxyl ions [50,51,52] and accumulate in the microchamber, causing a net decrease in pH over the 120 s experiment.

Comparisons between T-shaped and star-shaped devices revealed that the pH change in the star-shaped device is about 35% times greater, on average, than the T-shaped device over the frequencies tested. This demonstrated that the Faradaic reaction rates, which cause the medium’s pH changes, are larger in the star-shaped device compared with the T-shaped device, owing to the 60% greater electrode surface area in the star-shaped device.

Comparisons between devices with and without a hafnium dioxide dielectric coating revealed that the HfO_2_ layer decreased the rate of Faradaic reactions over the studied frequencies. The mechanism for this result is attributed to physical isolation of the electrodes, which reduces electron transfer between the electrolyte and electrodes, shifting the electric field to non-Faradaic charge transfer, in which electrons or charged particles do not move across the interface, and the current would be transferred by the electrical double-layer charge and discharge. Overall, the change in pH was reduced by an average of ~60% for star-shaped devices and ~30% for T-shaped devices coated with a hafnium oxide layer compared with uncoated devices at frequencies 0.2 $f_{c}$, 0.6 $f_{c}$, 1 $f_{c}$, and 1.4 $f_{c}$.

In summary, this work qualitatively and quantitatively characterizes pH gradients over time at a previously unexplored frequency range below, at, and above the electrode charging frequency. The rise and then fall in pH documented herein reveals hydronium and hydroxide ion behaviors at shorter and longer timescales, which will inform future electrokinetic microdevice development and operation. This work fills in a critical gap in the frequency parameter space. The implications are that ion artifacts can be more precisely controlled for or eliminated from chemical and biological analytical applications in lab-on-a-chip devices. This work also expanded the knowledge on the functional frequency range for HfO_2_ coatings, which are unable to remain structurally intact at 0.2 $f_{c}$. However, HfO_2_ remains a practical strategy to reduce electron transfer and prevent Faradic reactions.

## Figures and Tables

**Table 1:** Faradaic reaction detection in electrokinetic microfluidics involving different phenomena, including pH and ion gradients as well as RBC lysis and crenation.

Detected Phenomena	Medium	Charging Frequency, $f_{c}$ (kHz)	Applied Frequency	Characteristic Length (µm)	Reference
pH gradient	4 mM NaCl	3.3	0.9 $f_{c}$–5 $f_{c}$	100	[7]
pH gradient	0.1 mM KCl	3	0.167 $f_{c}$	10	[32]
pH gradient	0.1 mM KCl	3	0.167 $f_{c}$	20	[32]
pH gradient	0.1 mM KCl	0.6	0.167 $f_{c}$	80	[41]
Ion gradient	Methanol	20	5 $f_{c}$–25 $f_{c}$	100	[8]
RBC lysis	Phosphate buffer saline	2.79	0.36 $f_{c}$–1 $f_{c}$	50	[10]
RBC crenation	120, 145, 685 mM NaCl	17.8	14 $f_{c}$–72 $f_{c}$	100	[9]

**Table 2:** Conductivity and relative permittivity values used in the simulations.

	Conductivity (S/m)	Relative Permittivity
Buffer Solution ^1^	4.78 × 10^−2^	77.98
Hafnium Oxide ^2^	1 × 10^−16^	20.32
Gold ^3^	45.6 × 10^6^	6.9

1—set to match the conductivity in experiments; the relative permittivity of the buffer solution was assumed to be that of water. 2—obtained from [42]. 3—provided in COMSOL’s material library.

## Data Availability

To access the data supporting the findings of this study, please contact the corresponding author directly for further information.

## Supplementary Materials

The following supporting information can be downloaded at https://www.mdpi.com/article/10.3390/mi14091655/s1. Figure S1. Normalized intensity-pH calibration graph with whisker bars indicating standard deviation. The curve was obtained with a sigmoidal function with four parameters. (B) Comparison between the experimental data and the approximated data. The line y = x represents the data with zero percent error (blue) and the green lines correspond to the ±5 percent error. The graph shows that the approximation has less than 5 percent error for all. Figure S2. The photobleaching effect was monitored over 120 s for the standard solution pH of 7.7. Intensity, from a fixed exposure time of 308.4 ms, was normalized against the initial value to compare across experiments. Results indicate that photobleaching accounts for a 1.4% change over 120 seconds and was therefore neglected in further analysis.
