# Supplementary material for: pH Gradients in Spatially Non-Uniform AC Electric Fields around the Charging Frequency; A Study of Two Different Geometries and Electrode Passivation
Source: Micromachines (Basel). 2023 Aug 23;14(9):1655. doi: 10.3390/mi14091655 (PMC10534923; doi:10.3390/mi14091655)
Supplement: Supplementary file 1 [file micromachines-14-01655-s001.zip › micromachines-2518294-supplementary.pdf]

Supplemental Information

1. Normalized intensity-pH calibration plot:

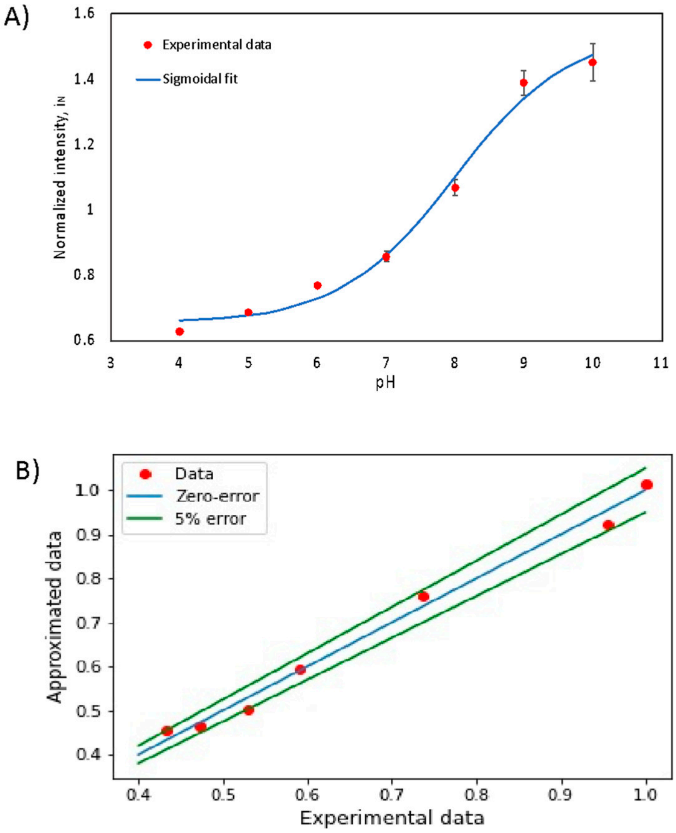

Figure S1. (**A**) Normalized intensity-pH calibration graph with whisker bars indicating standard deviation. The curve was obtained with a sigmoidal function with four parameters. (**B**) Comparison between the experimental data and the approximated data. The line y = x represents the data with zero percent error (blue) and the green lines correspond to the ±5 percent error. The graph shows that the approximation has less than 5 percent error for all.

2. The following figure shows the change in normalized intensity (normalized with respect to the intensity at time 0 s) over time. Repeats were conducted for a pH of 7.7. The decline from 1.000 to 0.986 can be attributed to FITC photobleaching.

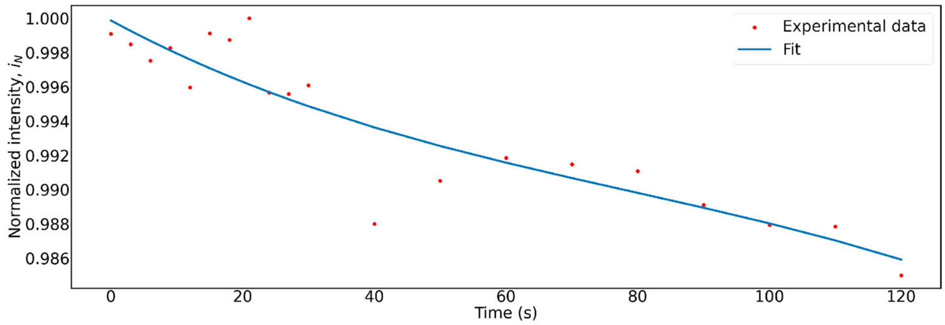

Figure S2. The photobleaching effect was monitored over 120 s for the standard solution pH of 7.7. Intensity, from a fixed exposure time of 308.4 ms, was normalized against the initial value to compare across experiments. Results indicate photobleaching accounts for a 1.4% change over 120 s and was therefore neglected in further analysis.
